# Multicenter Validation of Artificial Intelligence Predicting Anterior Circulation Large Vessel Occlusion Using Noncontrast Head CT

**DOI:** 10.1161/SVIN.125.001788

**Published:** 2025-07-30

**Authors:** Jong‐Won Chung, Myungjae Lee, Sue Young Ha, Pyeong Eun Kim, Leonard Sunwoo, Nakhoon Kim, Kwang‐Yeol Park, Kyu Sun Yum, Dong‐Ick Shin, Hong‐Kyun Park, Yong‐Jin Cho, Keun‐Sik Hong, Jae Guk Kim, Soo Joo Lee, Joon‐Tae Kim, Woo‐Keun Seo, Oh Young Bang, Gyeong‐Moon Kim, Dongmin Kim, Hee‐Joon Bae, Wi‐Sun Ryu, Beom Joon Kim

**Affiliations:** ^1^ Department of Neurology Samsung Medical Center Sungkyunkwan University School of Medicine Seoul Republic of Korea; ^2^ Artificial Intelligence Research Center JLK Inc. Seoul Republic of Korea; ^3^ Department of Radiology, College of Medicine Seoul National University Bundang Hospital Seongnam Republic of Korea; ^4^ Department of Neurology, Seoul National University College of Medicine Seoul National University Bundang Hospital Republic of Korea; ^5^ Department of Neurology Chung‐Ang University College of Medicine Chung‐Ang University Hospital Seoul Republic of Korea; ^6^ Department of Neurology College of Medicine Chungbuk National University Hospital Chungbuk National University Cheongju Republic of Korea; ^7^ Department of Neurology Inje University Ilsan Paik Hospital, InJe University College of Medicine Goyang Republic of Korea; ^8^ Department of Neurology Daejeon Eulji Medical Center School of Medicine Eulji University Daejeon Republic of Korea; ^9^ Department of Neurology Chonnam National University Hospital Chonnam National University Medical School Gwangju Republic of Korea

**Keywords:** artificial intelligence, computed tomography, ischemic stroke, large vessel occlusion

## Abstract

**Background:**

To validate an artificial intelligence software (JLK CTL) for predicting anterior circulation large vessel occlusion (LVO) using noncontrast computed tomography (NCCT) and to investigate its clinical implications regarding both infarct volume and outcomes.

**Methods:**

Between January 2021 and April 2023, we retrospectively included consecutive patients who concurrently underwent computed tomography angiography and NCCT within 24‐hour of last known well from 6 stroke centers. Additionally, 274 subjects without stroke were included in this study to evaluate the specificity of the software. The performance to identify LVO was evaluated based on the area under the receiver operating characteristic curve, as well as its sensitivity and specificity. The association between predicted JLK CTL LVO scores and infarct volumes and functional outcomes was assessed using Pearson correlation and logistic regression analyses, respectively.

**Results:**

Among 534 (mean age 69.9±13.2 years, 58.4% men) included patients, the median time from last known well to NCCT was 3.8 hours (interquartile range 1.7–9.5), with 30.7% (n = 164) presenting with LVO. The software demonstrated area under the receiver operating characteristic curve of 0.859 (95% CI, 0.827–0.887), with a sensitivity of 0.787 (95% CI, 0.716–0.847) and a specificity of 0.832 (95% CI, 0.790–0.869) at the predefined threshold. In subjects without ischemic stroke, the software achieved a specificity of 0.898 (95% CI, 0.887–0.922). The predicted JLK CTL LVO scores showed a correlation with infarct volumes on follow‐up diffusion‐weighted imaging (r = 0.54; *P*<0.001). After adjusting covariates, 1‐point increment of JLK CTL LVO score was associated with 2% increase of unfavorable 3‐month outcome (*P* = 0.011).

**Conclusion:**

In this multicenter study, we validated the performance of artificial intelligence software in predicting LVO on NCCT. Furthermore, the associations between JLK CTL LVO score and follow‐up infarct volume, as well as functional outcomes, support its clinical utility beyond merely screening patients who require rapid decision‐making.

**Nonstandard Abbreviations and Acronyms**
ASPECTS: Alberta Stroke Program Early CT Score
AUC‐ROC: area under the receiver operating characteristic curve
EVT: endovascular thrombectomy
ICA: internal carotid artery
LKW: last known well
LVO: large vessel occlusion
MCA: middle cerebral artery
mRS: modified Rankin scale
NCCT: noncontrast computed tomography
NIHSS: National Institutes of Health Stroke Scale

**Clinical Perspective**

**What Is New?**

- This is the first multicenter study to validate an artificial intelligence ‐based large vessel occlusion prediction model (JLK CTL) using only noncontrast computed tomography, demonstrating robust diagnostic accuracy with area under the receiver operating characteristic curve of 0.859 (95% CI, 0.827–0.887).
- The JLK CTL large vessel occlusion score, derived from an ExtraTrees ensemble algorithm, showed strong correlation with infarct volume and independently predicted poor 3‐month functional outcomes.

**What Are the Clinical Implications?**

- In real‐world emergency settings where computed tomography angiography is unavailable, delayed, or contraindicated, the JLK CTL large vessel occlusion score may assist clinicians in early detection of anterior circulation large vessel occlusion using only noncontrast computed tomography.
- This artificial intelligence‐driven tool may enable faster triage, guide timely transfer to thrombectomy‐capable centers, and support decision‐making when rapid vascular imaging is not feasible.

Anterior circulation large vessel occlusion (LVO) accounts for up to 46% of ischemic stroke^1^ and is associated with unfavorable outcome after ischemic stroke.^2^ Rapid and accurate diagnosis of LVO in the emergency department is essential for prompt intervention to mitigate further brain damage. However, patients who present with mild neurological deficits or atypical stroke symptoms are often misdiagnosed as having stroke mimics,^3^ posing a significant challenge to physicians who initially assess patients with neurological symptoms. Given the critical importance of early recognition of LVO for timely interventions and improved functional outcomes, there remains an unmet clinical need for an accurate and rapid LVO diagnosis.

Although noncontrast computed tomography (NCCT) scans are highly accessible and offer rapid acquisition, accurately diagnosing LVO from these scans requires expertise in interpreting NCCT scans and clinical correlation, which is often scarce in limited resources settings. Recent advances in artificial intelligence (AI) have facilitated the development of several software packages predicting LVO using NCCT.^4^, ^5^ In a recent study,^6^ an AI software (JLK CTL) that predicts anterior circulation LVO solely based on NCCT images by analyzing hemispheric differences in Hounsfield units (HU), regional brain volume, and the presence of a clot sign in the middle cerebral artery (MCA)^7^ was reported.

In this multicenter study involving 6 comprehensive stroke centers, we validated JLK CTL in patients who visited hospitals within 24 hours of their last known well (LKW). Furthermore, we investigated the clinical implications of JLK CTL LVO scores in terms of infarct‐relevant steno‐occlusion of the MCA, infarct volumes on follow‐up diffusion‐weighted imaging (DWI), and 3‐month functional outcomes after ischemic stroke.

## Methods

The data that support the findings of this study are available from the corresponding author upon reasonable request. Access to the data may be subject to institutional and ethical approvals, as well as data‐sharing agreements in accordance with applicable regulations.

### Study Populations

This multicenter study originated from a brain imaging substudy of the ongoing nationwide stroke registry, CRCS‐K (Clinical Research Collaboration for Stroke in Korea).^8^ We consecutively enrolled patients with ischemic stroke or transient ischemic attack who admitted within 7 days of symptom onset from April 2022 to April 2023 at 5 comprehensive stroke centers (Figure S1). To ensure the heterogeneity of the data, we additionally collected a consecutive series of patients between January 2021 and March 2022 from another stroke center, which did not participate in the CRCS‐K stroke registry. Inclusion criteria were (1) aged 18 years or older, and (2) concurrently underwent NCCT and CT angiography. Exclusion criteria were (1) NCCT performed beyond 24 hours of LKW; (2) posterior circulation stroke; (3) poor image quality to analyze; (4) NCCT with contrast; (5) cases with any hemorrhage, brain tumor, or external ventricular drain; (6) chronic anterior circulation LVO; and (7) NCCT acquired after endovascular thrombectomy (EVT). Additionally, to evaluate the false positive rate of the software system in individuals without stroke, we prospectively enrolled a consecutive cohort of 274 patients from a separate stroke center. These patients presented to the emergency department with suspected strokes between February and May 2023 but were ultimately determined to be stroke free based on follow‐up DWI scans. All image data were collected at the central image lab and analyzed independently of the software provider. All patients or their legal representatives gave written informed consent. The study protocol was approved by the institutional review board of Seoul National University Bundang Hospital [B‐2307‐841‐303]. All clinical and imaging data were anonymized prior to data collection. This study adheres to the Standards for Reporting of Diagnostic Accuracy Studies guidelines.

### Clinical Data Collection

We retrieved baseline demographic and clinical information for all study participants from a web‐based prospective stroke cohort (strokedb.or.kr) or electronic medical records.^9^ This included age, sex, history of previous stroke, functional status before stroke, and cardiovascular risk factors such as hypertension, diabetes, and atrial fibrillation. Stroke characteristics included the time interval between LKW and NCCT scan, the National Institutes of Health Stroke Scale (NIHSS) score at admission, and treatment information. Functional status at 3 months post stroke was measured using the modified Rankin Scale (mRS) score, determined through a structured telephone interview by an experienced physician assistant at each hospital as previously described.^10^, ^11^

### CT Imaging Protocols and Analysis

NCCT scans were acquired according to standard departmental protocols in each hospital. Slice thickness ranged from 3 to 5 mm (Table S1). In the present study, LVO was defined as an arterial occlusion encompassing the intracranial segment of the internal carotid artery (ICA), as well as the M1 and proximal M2 segments of the MCA (MCA‐M1 and MCA‐M2, respectively).^12^ To confirm the presence of anterior circulation LVO, CT angiography source images, maximum intensity projection images, and 3‐dimensional rendering images that were concurrently performed with NCCT were thoroughly examined by 2 experienced vascular neurologists, alongside an evaluation of patients’ follow‐up magnetic resonance imaging scans and symptomatic data. In cases of diagnostic discrepancy, a final determination was made by an experienced neuroradiologist. Along with the presence of LVO, location (ICA, MCA‐M1, and MCA‐M2) and the laterality of LVO were recorded. If the LVO prediction by JLK CTL was correct but the laterality of the LVO was discordant with the experts’ consensus, we designated the case as a false negative. Isolated MCA‐M2 occlusion was defined as the presence of MCA‐M2 occlusion without concurrent ICA or MCA‐M1 occlusion. Infarct‐relevant MCA stenosis is defined as moderate to severe stenosis on CT angiography that is related to infarcts observed on DWIs. For each NCCT scan, an experienced neurologist rated Alberta Stroke Program Early CT Score (ASPECTS).^13^, ^14^ To evaluate the performance of expert neuroradiologists in detecting LVO on NCCT scans, we randomly selected a data set of 65 NCCT scans (35 LVO‐positive and 30 LVO‐negative). Two radiologists, each with >10 years of NCCT interpretation experience, independently reviewed the images to determine the presence of LVO while blinded to clinical information.

### AI Software

NCCT scans were processed through the validated AI software system (JLK CTL, JLK Inc., Republic of Korea) to predict LVO.^6^ In brief, the software analyzed differences in volume, tissue density, and HU distribution between bihemispheric regions (striatocapsular, insula, M1–M3, and M4–M6, modified from the ASPECTS).^13^, ^14^ Additionally, the deep learning algorithm also automatically segmented hyperdense MCA sign as an extra feature.^7^ An ExtraTrees (Extremely Randomized Trees) machine learning algorithm—an ensemble method using multiple randomized decision trees to improve prediction accuracy—was employed to predict the JLK CTL LVO score based on these features, which represents the likelihood of LVO determined by the algorithm ranging from 0 to 100. To determine the performance metric, we set the optimal threshold of the JLK CTL LVO score at 12.0, ensuring a sensitivity >70% while also maximizing specificity (Figure S2). To further assess the feasibility of real‐world implementation, we conducted prospective on‐site testing using a graphics processing unit‐equipped desktop in an emergency department setting.

### Follow‐Up Imaging Analysis

Follow‐up DWI within 7 days after NCCT was included to analyze the association between JLK CTL LVO score and follow‐up infarct volumes. If the patient underwent ≥2 DWIs, we analyzed the first image. Infarct volumes on DWI were calculated using a validated software package (JLK DWI, JLK Inc., Republic of Korea).^15^, ^16^, ^17^ The segmentation of the infarct area was carefully reviewed by an experienced vascular neurologist. To ensure accurate infarct segmentation, manual corrections were applied if required.

### Categorization of JLK CTL LVO Scores

We divided patients into deciles based on their JLK CTL LVO scores, with each decile representing 10% intervals. For each decile, we calculated the observed frequency of LVO determined by expert consensus. Based on a combination of the observed frequency of LVO and the distribution of patients across the deciles, we categorized the patients into 4 groups to ensure meaningful stratification.

### Statistical Analysis

Baseline characteristics among participating centers were compared using ANOVA or the Kruskal–Wallis test for continuous variables, and the chi‐square test for categorical variables, as appropriate. To validate the accuracy of the JLK CTL software in predicting LVO, we calculated the area under the receiver operating characteristic curve (AUC‐ROC), along with sensitivity, specificity, positive predictive value, and negative predictive value at the predefined threshold used in the previous study.^6^ We then compared the diagnostic accuracy of JLK CTL, ASPECTS, NIHSS, and the combined approach (JLK CTL + NIHSS) using their respective AUC‐ROCs. We used a 1000‐repeat bootstrap method to determine 95% CIs and compared AUC‐ROCs using the DeLong method.^18^ Additionally, we conducted the AUC‐ROC analysis after stratifying patients by participating centers. For the subgroup analysis, we performed additional analyses stratifying the data by the site of occlusion (ICA, MCA‐M1, and MCA‐M2) and by sex.

After stratifying patients into 4 subgroups—proximal LVO, isolated MCA‐M2 occlusion, infarct‐relevant MCA stenosis, and no steno‐occlusion—we compared JLK CTL LVO scores using ANOVA with Tukey's multiple comparison test. The association between JLK CTL LVO scores and infarct volumes on DWI was analyzed using Pearson correlation analysis. Furthermore, we analyzed the association between JLK CTL LVO scores and 3‐month mRS scores using ANOVA and multivariable ordinal logistic and binary (mRS score 0–2 versus 3–6) logistic regression analyses. Based on prior literature on functional outcomes after ischemic stroke,^11^, ^19^, ^20^, ^21^ age, sex, admission NIHSS score, previous stroke, hypertension, diabetes, atrial fibrillation, revascularization therapy, and time from LKW to NCCT scan were used as covariates. After stratification by EVT, the association between JLK CTL LVO score and binary outcomes were reanalyzed. All statistical analyses were performed using STATA software (version 16.0, College Station, TX, USA) and MedCalc (version 17.2, MedCalc Software, Ostend, Belgium). A *P* value<0.05 was considered statistically significant.

## Results

### Baseline Characteristics

Among the 1391 patients screened, 957 underwent concurrent NCCT and CT angiography. After excluding 423 patients, we included 534 patients in our analyses (Figure S1). The mean age of the included patients was 69.9 years (SD 13.2), and 312 (58.4%) were male. The median interval between LKW to NCCT scan was 3.8 hours (interquartile range 1.7–9.5) and 164 patients (30.7%) had LVO. Demographic and risk factor profiles were generally similar across participating centers, except for the prior history of stroke (Table 1). However, there were significant differences among the centers in terms of imaging vendors, time from LKW to NCCT scan, frequency of revascularization therapy, and the interval between NCCT and DWI (Table 1 and Table S1).

**Table 1:** Baseline Characteristics of Study Population

	All (N = 534)	Hospital A (n = 73)	Hospital B (n = 97)	Hospital C (n = 53)	Hospital D (n = 65)	Hospital E (n = 70)	Hospital F (n = 176)	*P* value
Age, y	69.9 ± 13.2	70.6 ± 14.1	70.3 ± 13.1	71.5 ± 13.0	69.3 ± 12.1	69.0 ± 15.2	69.4 ± 12.3	0.83
Sex, male	312 (58.4%)	40 (54.8%)	62 (63.9%)	29 (54.7%)	35 (53.9%)	41 (58.6%)	105 (60.0%)	0.77
Large vessel occlusion	164 (30.7%)	17 (23.3%)	32 (33.0%)	20 (37.7%)	21 (32.3%)	20 (28.6%)	54 (30.7%)	0.61
MCA M2 occlusion	40 (9.2%)	6 (8.2%)	5 (5.2%)	2 (3.8%)	5 (7.7%)	6 (8.6%)	16 (9.1%)	0.48
NIHSS score at admission	4 (1–11)	3 (0–10)	4 (1–11)	5 (2–11)	5 (1–13)	5 (2–14)	4 (1–11)	0.16^*^
Previous stroke	103 (19.3%)	10 (13.7%)	14 (14.4%)	17 (32.1%)	17 (26.2%)	8 (11.4%)	37 (21.0%)	0.018
Hypertension	351 (65.7%)	46 (63.0%)	62 (63.9%)	40 (75.5%)	45 (69.2%)	54 (77.1%)	104 (59.1%)	0.063
Diabetes	163 (30.5%)	16 (21.9%)	35 (36.1%)	16 (30.2%)	18 (27.7%)	20 (28.6%)	58 (33.0%)	0.44
Atrial fibrillation	139 (26.0%)	23 (31.5%)	24 (24.7%)	18 (34.0%)	18 (27.7%)	20 (28.6%)	36 (20.5%)	0.29
CT vendor								<0.001
Philips	75 (14.0%)	73 (100.0%)	2 (2.1%)	0	0	0	0	
GE medical systems	270 (50.6%)	0	95 (97.9%)	0	0	0	175 (99.6%)	
SIEMENS	116 (21.7%)	0	0	53 (100.0%)	63 (96.9%)	0	0	
Toshiba	73 (13.7%)	0	0	0	2 (3.1%)	70 (100.0%)	1 (0.4%)	
LKW to CT, hour	3.8 (1.7–9.5)	3.2 (1.4–10.0)	2.6 (1.4–4.8)	5.2 (1.9–9.6)	1.6 (1.2–2.4)	2.6 (1.6–5.9)	8.1 (3.8–13.9)	<0.001^*^
Revascularization therapy								<0.001
Intravenous only	42 (7.9%)	3 (4.1%)	9 (9.3%)	6 (11.3%)	6 (9.2%)	10 (14.3%)	8 (4.6%)	
Endovascular thrombectomy only	65 (12.2%)	8 (11.0%)	11 (11.3%)	5 (9.4%)	12 (18.5%)	7 (10.0%)	22 (12.6%)	
Combined	45 (8.5%)	10 (13.7%)	16 (16.5%)	3 (5.7%)	5 (7.7%)	5 (7.1%)	6 (3.5%)	
Interval between NCCT and DWI, h	1.6 (0.9–3.3)	2.3 (1.3–9.0)	0.7 (0.3–3.2)	0.6 (0.3–1.5)	1.7 (1.3–4.2)	2.0 (1.3–12.6)	1.8 (1.2–2.8)	<0.001^*^
Infarct volume on DWI, mL	3.7 (0.6–26.4)	12.3 (1.7–40.2)	2.5 (0.6–14.3)	9.5 (1.6–51.1)	0.7 (0.2–3.6)	1.5 (0.3–9.1)	6.8 (0.9–35.9)	<0.001^*^

Data are presented as mean±SD, number (percentage), and median (interquartile range).

CT indicates computed tomography; DWI, diffusion‐weighted imaging; LKW, last known well; MCA, middle cerebral artery; NCCT, noncontrast computed tomography; and NIHSS, National Institutes of Health Stroke Scale.

^*^ Kruskal–Wallis test was used.

### Efficacy of JLK CTL in a Multicenter Data Set

**Figure 1:**
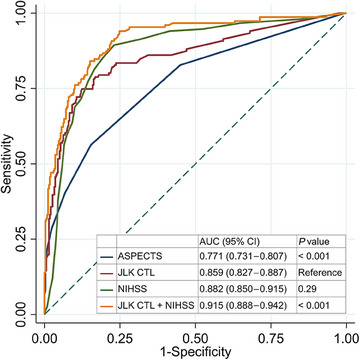
Receiver operating characteristic curves comparing JLK CTL, ASPECTS, NIHSS score, and JLK CTL+NIHSS scores for predicting large vessel occlusion using noncontrast brain CT scans. ASPECTS indicates Alberta Stroke Program Early CT score; AUC, area under the curve; and NIHSS, National Institutes of Health Stroke Scale.

Overall, JLK CTL achieved an AUC‐ROC of 0.859 (95% CI, 0.827–0.887), which was significantly higher than that of ASPECTS (0.771 [95% CI, 0.731–0.807]; *P*<0.001) and comparable to that of NIHSS (0.882 [95% CI, 0.850–0.915]; *P* = 0.29; Figure 1). Furthermore, combining JLK CTL with NIHSS produced superior results compared with either method alone (both *P*<0.001). At the JLK CTL LVO score threshold of 12.0, JLK CTL demonstrated a sensitivity of 0.787 (95% CI, 0.641–0.775), specificity of 0.832 (95% CI, 0.790–0.869), positive predictive value of 0.675 (95% CI, 0.604–0.741), and negative predictive value of 0.898 (95% CI, 0.861–0.928; Table 2). When stratified by time from LKW to NCCT scans (<6 versus 6–24 hours), JLK CTL exhibited comparable performance in both groups with AUC‐ROC of 0.843 and 0.984, respectively (Table 2). The performance of the JLK CTL varied across participating centers, with AUC‐ROCs ranging from 0.781 to 0.916 (Figure S3), sensitivity from 0.700 to 0.900, and specificity from 0.727 to 0.946 (Table 3). After stratifying by the site of occlusion, the algorithm's sensitivity was higher for proximal vessel occlusions than for distal MCA occlusions. The algorithm showed comparable performance in both men and women (Tables S2 and S3). The most common cause of false positives was chronic infarct, followed by calcification of the MCA. Additionally, relevant artery stenosis, distal vessel occlusion (including M3 or M4 segments of the MCA), and large infarct without LVO were also common presumed causes (Table S4).

**Table 2:** Performance of Artificial Intelligence Software in the Entire Population and Subgroups Stratified by Time From Last Known Well to Noncontrast CT Scans (<6 Versus 6–24 Hours) at the Predefined Threshold

Confusion matrix	All patients (N = 534)		LKW to NCCT<6 hours (n = 337)		LKW to NCCT>6 and ≤24 hours (n = 197)	
	Prediction		Prediction		Prediction	
	LVO	No LVO	LVO	No LVO	LVO	No LVO
Ground truth, LVO	129	35	95	27	34	8
Ground truth, no LVO	62	308	38	177	24	131
Sensitivity (95% CI)	0.787 (0.716–0.847)		0.779 (0.695–0.849)		0.810 (0.659–0.914)	
Specificity (95% CI)	0.832 (0.790–0.869)		0.823 (0.766–0.872)		0.845 (0.778–0.898)	
Positive predictive value (95% CI)	0.675 (0.604–0.741)		0.714 (0.630–0.789)		0.586 (0.449–0.714)	
Negative predictive value (95% CI)	0.898 (0.861–0.928)		0.868 (0.813–0.911)		0.942 (0.890–0.975)	
AUC‐ROC	0.859 (0.827–0.887)		0.843 (0.800–0.881)		0.894 (0.843–0.934)	

AUC‐ROC indicates area under the receiver operating characteristics curve; CT, computed tomography; LKW, last known well; LVO, large vessel occlusion; NCCT, noncontrast computed tomography.

**Table 3:** Performance of Artificial Intelligence Software in Each Participating Center

Confusion matrix	Hospital A (n = 73)		Hospital B (n = 97)		Hospital C (n = 53)		Hospital D (n = 65)		Hospital E (n = 70)		Hospital F (n = 176)	
	AI prediction		AI prediction		AI prediction		AI prediction		AI prediction		AI prediction	
	LVO	No LVO	LVO	No LVO	LVO	No LVO	LVO	No LVO	LVO	No LVO	LVO	No LVO
Ground truth, LVO	12	5	25	7	18	2	17	4	14	6	43	11
Ground truth, no LVO	3	53	14	51	9	24	8	36	10	40	18	104
Sensitivity (95% CI)	0.706 (0.440–0.897)		0.781 (0.600–0.907)		0.900 (0.683–0.988)		0.810 (0.581–0.946)		0.700 (0.457–0.881)		0.796 (0.665–0.894)	
Specificity (95% CI)	0.946 (0.851–0.989)		0.785 (0.665–0.877)		0.727 (0.545–0.867)		0.818 (0.673–0.918)		0.800 (0.663–0.900)		0.852 (0.777–0.910)	
Positive predictive value (95% CI)	0.800 (0.519–0.957)		0.641 (0.472–0.788)		0.667 (0.460–0.835)		0.680 (0.465–0.851)		0.583 (0.366–0.779)		0.705 (0.574–0.815)	
Negative predictive value (95% CI)	0.914 (0.810–0.971)		0.879 (0.767–0.950)		0.923 (0.749–0.991)		0.900 (0.763–0.972)		0.870 (0.737–0.951)		0.904 (0.835–0.951)	
AUC‐ROC	0.781 (0.669–0.869)		0.790 (0.696–0.866)		0.915 (0.806–0.974)		0.916 (0.820–0.970)		0.793 (0.679–0.881)		0.897 (0.842–0.938)	

AI indicates artificial intelligence; AUC‐ROC, area under the receiver operating characteristics curve; LVO, large vessel occlusion.

Of 274 subjects without ischemic stroke enrolled from a single stroke center, JLK CTL showed specificity of 0.898 (95% CI, 0.887–0.922), with consistent specificity across difference vendors (Table S5).

In randomly selected 65 NCCT scans, rater 1 and rater 2 achieved sensitivity of 51.4% (95% CI, 34.0%–68.6%) and 57.1% (39.4%–73.7%) and specificity of 93.3% (77.9%–99.2%) and 83.3% (65.2–94.4%), respectively. The interrater reliability (kappa) was 0.69 (95% CI, 0.52–0.88). In the same data set, JLK CTL achieved a sensitivity of 77.1% (59.9%–89.6%) and a specificity of 93.3% (77.9%–99.2%).

On randomly selected 100 NCCT scans with slice thickness of 5 mm, JLK CTL showed inference time of 95±25 seconds. The inference time includes uploading and reading input data, running machine learning modules, postprocessing and visualization of output. The test was conducted on Nvidia RXT 2080Ti GPU, Intel Core i5‐12600K CPU, and 16GB of RAM. In 100 consecutive patients presenting to a university hospital emergency department, the algorithm demonstrated a mean inference time of 104±12 seconds.

### Categorizing JLK CTL LVO Scores Using Observed Frequency of LVO in Multicenter Data

When patients were stratified into deciles based on their JLK CTL LVO scores, a nearly linear increase in LVO frequency was observed with higher scores (Figure S4A). However, due to the small number of patients in groups with JLK CTL LVO scores>40, interpretations from these groups may be less reliable. Therefore, we categorized JLK CTL LVO scores into 4 groups: 0–10 (unlikely), 11–20 (less likely), 21–50 (possible), and 51–100 (suggestive), considering both the patient distribution and observed LVO frequencies. After categorization, the observed frequencies of LVO were 9.8%, 23.1%, 71.6%, and 91.7% in unlikely, less likely, possible, and suggestive groups, respectively (Figure S4B).

### Associations of JLK CTL LVO Scores With Steno‐Occlusion of Middle Cerebral Artery and Infarct Volumes on Follow‐Up Diffusion‐Weighted Imaging

**Figure 2:**
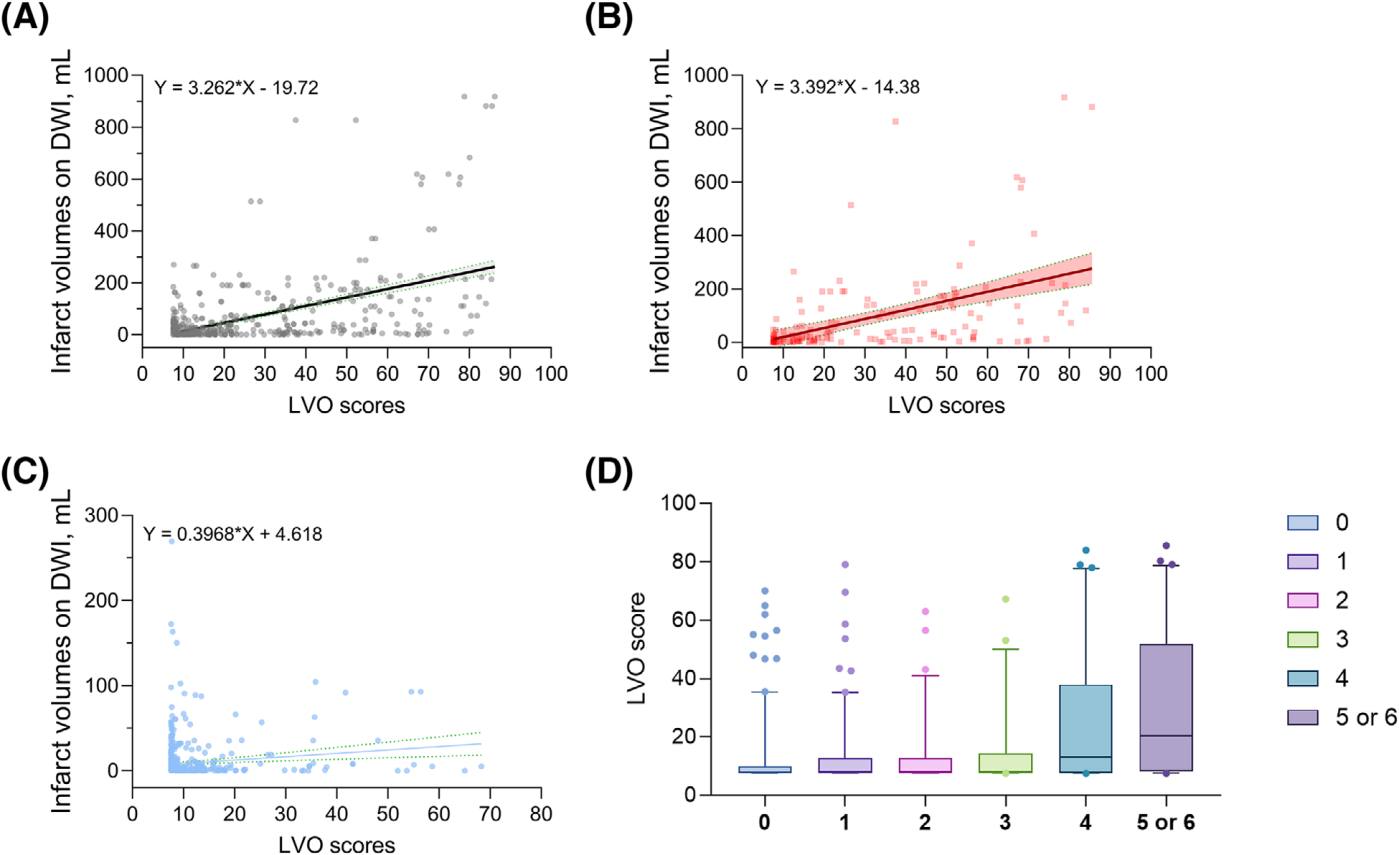
Dot plots illustrate the relationship between JLK CTL large vessel occlusion scores and infarct volumes on follow‐up diffusion‐weighted imaging in (**A**) all patients, (**B**) patients with LVO, and (**C**) patients without LVO. The black, red, and blue lines with shaded areas represent linear regression lines and their 95% CIs for patients with and without LVO, respectively. **D** Box plot showing JLK CTL LVO scores stratified by modified Rankin scale score at 3 months after ischemic stroke. DWI indicates diffiusion‐weighted imaging; and LVO, large vessel occlusion.

In comparison to other groups, the LVO group (n = 128) exhibited a higher JLK CTL LVO score, with a mean±SD of 35.1 (23.4; Table 4). In addition, the isolated MCA‐M2 occlusion group (n = 36) had higher JLK CTL LVO scores (21.9±18.1) compared with the group with infarct‐relevant MCA stenosis (n = 36; 9.8±4.9) and those without steno‐occlusion of the MCA (n = 334; 10.2±7.4). DWIs were available for 485 patients with a median interval of 1.6 hours (interquartile range 0.9–3.3) from the NCCT scans. JLK CTL LVO scores were significantly correlated with infarct volumes on follow‐up DWI (r = 0.54; *P*<0.001; Figure 2A). This correlation was stronger in patients with LVO (n = 150; r = 0.47; Figure 2B) compared with those without LVO (n = 335; r = 0.09; Figure 2C).

**Table 4:** JLK CTL LVO Scores Across Subgroups

Subgroup	JLK CTL LVO score	*P* value^*^	*P* value^†^	*P* value^‡^
No steno‐occlusion (n = 334)	10.2 ± 7.4	Reference		
Relevant artery stenosis (n = 36)	9.8 ± 4.9	0.99	Reference	
Isolated MCA occlusion (n = 36)	21.9 ± 18.1	0.002	0.013	Reference
Large vessel occlusion (n = 128)	35.1 ± 23.4	<0.001	<0.001	0.012

Statistical comparisons were conducted using ANOVA followed by Tukey post‐hoc analysis. LVO indicates large vessel occlusion; and MCA, middle cerebral artery.

^*^ *P* value for comparison with the no steno‐occlusion group.

^†^ *P* value for comparison with the no relevant artery stenosis group.

^‡^ *P* value for comparison with the isolated MCA occlusion group.

### JLK CTL LVO Scores and Functional Outcomes

JLK CTL LVO scores were significantly associated with 3‐month mRS scores after ischemic stroke (*P*<0.001; Figure 2D). Multivariable ordinal and binary logistic regression analyses confirmed an independent relationship between JLK CTL LVO scores and 3‐month functional outcomes after ischemic stroke (Table S6). After adjusting for covariates, each 1‐point increase in the JLK CTL LVO score was associated with a 2% higher likelihood of worse mRS scores or unfavorable outcomes. After stratifying by EVT status, the JLK CTL LVO score was independently associated with unfavorable outcomes in patients who underwent EVT, whereas no such association was observed in those who did not receive EVT (Table S7).

## Discussion

In this multicenter study using data from multiple centers, we validated JLK CTL, a software designed to predict anterior circulation LVO on NCCT. JLK CTL demonstrated a sensitivity of 0.787 and a specificity of 0.832 at the predefined threshold. Across hospitals with different imaging vendors and varying LVO prevalence, JLK CTL consistently maintained a sensitivity >70%. Additionally, JLK CTL LVO scores correlated with infarct volumes in patients with LVO. Moreover, an increase in the JLK CTL LVO score was associated with a 2% higher likelihood of worse mRS scores.

In a recent study involving 1453 patients from 2 stroke centers, a deep learning algorithm (MethinksLVO, Methinks Software S.L., Spain) demonstrated an AUC‐ROC of 0.87 (sensitivity: 83%, specificity: 71%) for LVO detection on NCCT,^5^ which is comparable to the performance in our study. However, their algorithm employed convolutional neural networks, which are not capable of explaining the rationale behind their conclusions.^22^ In contrast, our algorithm uses machine learning based on processed image features commonly used in clinical practice, allowing it to provide explainable results to physicians. For instance, our model highlights in red those areas within the modified ASPECTS region where HU values significantly decrease. In addition, JLK CTL displays a feature‐importance graph indicating which features most strongly increase or decrease the probability of LVO (see Figure S5). Additionally, a study involving 244 cases, including 115 with LVO, demonstrated that the RAPID NCCT Stroke (RAPID, iSchemaView, Menlo Park, CA) algorithm had a sensitivity of 63.5% and a specificity of 95.1% for the prediction of LVO.^4^ In addition to the suboptimal sensitivity of the algorithm, the algorithm relied solely on the hyperdense vessel sign for LVO detection, limiting its applicability to ethnicities where cardioembolism is less frequently the cause of LVO, such as in Asian and Black populations.^23^, ^24^

During the evaluation of JLK CTL across participating centers, we observed variability in performance metrics. Despite comparable baseline characteristics among stroke centers and the absence of significant associations between JLK CTL performance and factors such as time from LKW to NCCT scan or revascularization rates, we hypothesize that inherent overfitting of the AI model may underlie these discrepancies.^25^, ^26^ Given that our algorithm primarily relies on HU differences between hemispheres as a key feature, interscanner variability in HU calibration across different imaging systems^27^, ^28^, ^29^ may contribute to the observed inconsistencies in performance. Nevertheless, irrespective of scanner vendor or hospital, the algorithm consistently demonstrated sensitivity and specificity >70%, indicating its potential utility as a screening tool for identifying patients who may require further evaluation or transfer to an EVT‐capable center. Additionally, the high specificity (89.8%) in subjects without ischemic stroke further supports the utility of JLK CTL as a screening tool for patients with suspected ischemic stroke.

We found that patients with isolated MCA‐M2 occlusion had significantly higher JLK CTL LVO scores compared with those with infarct‐relevant MCA stenosis or without steno‐occlusion of the MCA. This finding suggests that the algorithm could be used for both proximal and more distal MCA occlusions. Because the algorithm uses ASPECTS regions M1–M3 and M4–M6 as radiomics features, it was able to detect isolated MCA‐M2 occlusions, although their JLK CTL LVO scores were lower than those for acute proximal LVO.

ASPECTS is negatively associated with infarct volume on DWI.^30^ However, inconsistent results have been reported regarding the association between ASPECTS on baseline NCCT and functional outcomes.^31^, ^32^ Despite a very short time interval between LKW to NCCT scans (median 3.8 hours) in the present study, we observed a significant correlation between the JLK CTL LVO score and infarct volume on DWI that was obtained with a median interval of 1.6 hours between NCCT scans and DWI. In addition, we found that JLK CTL LVO score was associated with unfavorable outcomes after ischemic stroke. If warranted in future studies, the JLK CTL LVO score could become a radiomic prognosticator for patients with ischemic stroke.

The JLK CTL LVO score reflects both the extent and severity of hypoattenuation, aligning closely with the volume and severity of ischemic lesions. This capability offers the potential to establish thresholds for clinical decision‐making, helping to identify cutoff values where risks such as hemorrhagic transformation or edema^33^ may outweigh the benefits of thrombolytic therapy.

Real‐world deployment of AI‐based stroke detection requires not only accuracy but also efficiency in diverse clinical settings. Our study demonstrated a mean inference time of 95 seconds on a standard GPU‐equipped desktop and 104±12 seconds in an emergency department setting, supporting its feasibility in hospitals. However, implementation in mobile stroke units and resource‐limited settings may be affected by hardware constraints, network dependency, and processing delays. Although on‐site processing enables real‐time decision support, cloud‐based deployment offers scalability but may introduce latency. Future work should focus on model optimization and hardware adaptation to enhance real‐world applicability.

We observed notable intercenter variability in our algorithm's performance, which may be attributed to differences in HU calibration and image acquisition parameters across various scanners.^34^ Although we did not incorporate scanner‐dependent HU normalization or standardization in the present study, we recognize that such strategies could reduce this variability and enhance the software's accuracy. Moving forward, further investigation into the feasibility of scanner‐specific standardization techniques—such as vendor‐based calibration tables or reference phantoms—will be warranted to mitigate inter‐scanner discrepancies. By implementing these measures, we anticipate further improvements in the robustness and generalizability of our algorithm for routine clinical practice.

Our study has limitations. First, we included only an Asian population with stroke. The cause of LVO differs between races or ethnicities,^23^, ^24^ which could potentially affect the software's performance. Future studies should include multinational, multiethnic populations to validate the algorithm's generalizability. Second, JLK CTL can detect only ICA or MCA occlusion and thus is not applicable to anterior cerebral artery, posterior cerebral artery, and posterior circulation LVO. More important, although we validated the algorithm in patients without ischemic stroke who underwent both NCCT and DWI scans, the algorithm was not tested in real‐world clinical practice, where the prevalence of ischemic stroke varies across different clinical settings. Third, DWI images were obtained soon after the NCCT, which may imply a lower correlation with the infarct volume because it is not fully established at that early stage. However, this short interval may bias the association between the JLK CTL LVO score and infarct volume toward the null. Fourth, to further facilitate stroke care, the applicability of JLK CTL on mobile stroke units should be considered. This aspect will need to be validated in future research to fully assess the potential of our software in improving stroke care. Lastly, although our findings support the feasibility of implementing JLK CTL in standard hospital environments, deployment in low‐resource or rural settings may be limited by hardware availability, internet connectivity, and infrastructure for image processing. These challenges should be considered in future efforts to scale the software globally.

In conclusion, we demonstrated the clinical efficacy of JLK CTL in predicting LVO on NCCT using a multicenter data set. Additionally, JLK CTL LVO scores correlated with infarct volumes on follow‐up DWI and 3‐month functional outcomes after ischemic stroke. These findings suggest that JLK CTL LVO scores could serve as a valuable prognosticator in future research endeavors. The software may assist physicians in rapidly identifying patients with stroke who require further investigation and treatment.

## Disclosure

Myungjae Lee, Sue Young Ha, Pyeong Eun Kim, Dongmin Kim, and Wi‐Sun Ryu are employees of JLK Inc., Seoul, Republic of Korea.

## Sources of Funding

This research was supported by the Multiministry Grant for Medical Device Development (KMDF_PR_20200901_0098), funded by the Korean government and a grant of the Korea Health Technology R&D Project through the Korea Health Industry Development Institute, and funded by the Ministry of Health & Welfare, Republic of Korea (grant number: HI22C0454).

## Supporting information




**Figure S1**: Study flow chart
**Figure S2**: Change in sensitivity and specificity of JLK‐CTL depending on different thresholds
**Figure S3**: Receiver operating characteristics curves in each participating center using JLK‐CTL
**Figure S4**: Predicted and observed probabilities of large vessel occlusion (LVO) after stratifying deciles of LVO score and categorized LVO scores
**Figure S5**: Representative case showing the results of JLK‐CTL
**Table S1**: Noncontrast CT and CT angiography parameters in participating centers
**Table S2**: Sensitivity of the algorithm after stratification by the site of occlusion
**Table S3**: Performance of the algorithm after stratification by sex
**Table S4**: Details of cases with false positives by the algorithm
**Table S5**: Specificity of artificial intelligence software by different computed tomography vendors and models in subjects without ischemic stroke
**Table S6**: Multivariable ordinal and binary logistic regression analysis between 3‐month functional outcome and JLK‐CTL large vessel occlusion scores
**Table S7**: Multivariable binary logistic regression analysis between binary unfavorable 3‐month outcome and JLK CTL large vessel occlusion scores after stratification by endovascular thrombectomy

Supporting Information
