# Supplementary material for: Multicenter Validation of Artificial Intelligence Predicting Anterior Circulation Large Vessel Occlusion Using Noncontrast Head CT
Source: Stroke Vasc Interv Neurol. 2025 Jul 30;5(5):e001788. doi: 10.1161/SVIN.125.001788 (PMC12697659; doi:10.1161/SVIN.125.001788)
Supplement: Supplementary file 1 — Figure S1: Study flow chart Figure S2: Change in sensitivity and specificity of JLK‐CTL depending on different thresholds Figure S3: Receiver operating characteristics curves in each participating center using JLK‐CTL Figure S4: Predicted and observed probabilities of large vessel occlusion (LVO) after stratifying deciles of LVO score and categorized LVO scores Figure S5: Representative case showing the results of JLK‐CTL Table S1: Noncontrast CT and CT angiography parameters in participating centers Table S2: Sensitivity of the algorithm after stratification by the site of occlusion Table S3: Performance of the algorithm after stratification by sex Table S4: Details of cases with false positives by the algorithm Table S5: Specificity of artificial intelligence software by different computed tomography vendors and models in subjects without ischemic stroke Table S6: Multivariable ordinal and binary logistic regression analysis between 3‐month functional outcome and JLK‐CTL large vessel occlusion scores Table S7: Multivariable binary logistic regression analysis between binary unfavorable 3‐month outcome and JLK CTL large vessel occlusion scores after stratification by endovascular thrombectomy [file SVI2-5-e001788-s002.pdf]

Supplemental Materials

Multicenter validation of artificial intelligence predicting anterior circulation large vessel occlusion using noncontrast head CT

Chung et al.

Supplemental Figure 1. Study flow chart

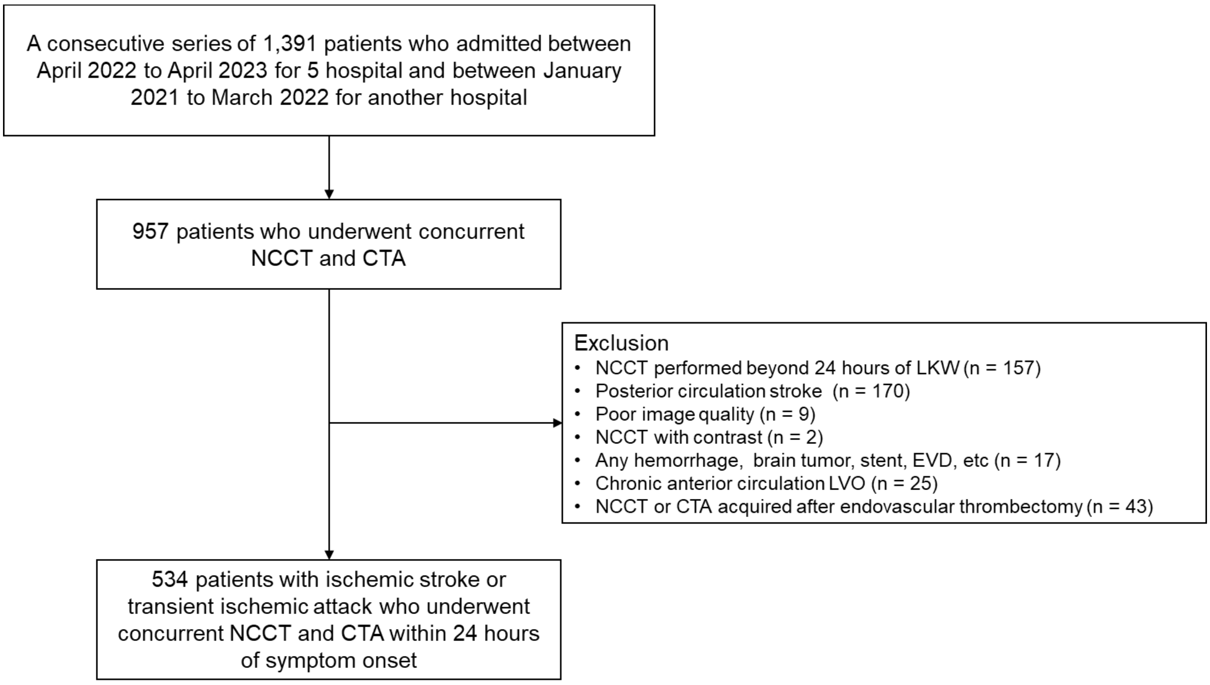

NCCT=noncontrast CT; CTA=CT angiogram; LKW=last known well; EVD=extraventricular drainage.

Supplemental Figure 2. Change in sensitivity and specificity of JLK-CTL depending on different thresholds

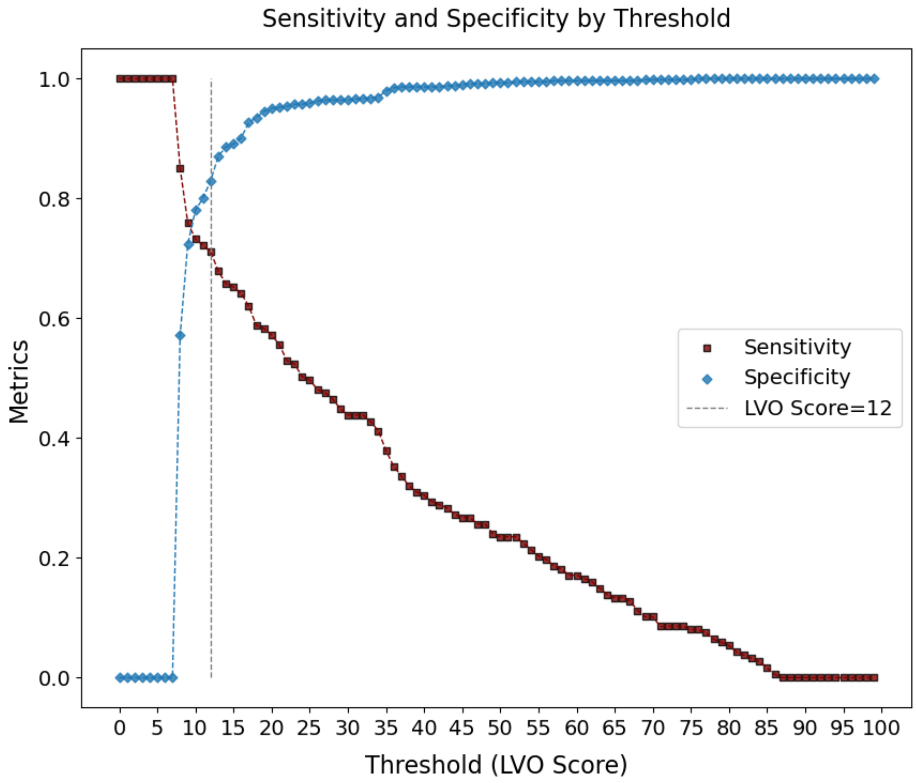

Thresholds were increased from 0 to 100 with an interval of 2.

Supplemental Figure 3. Receiver operating characteristics curves in each participating center using JLK-CTL

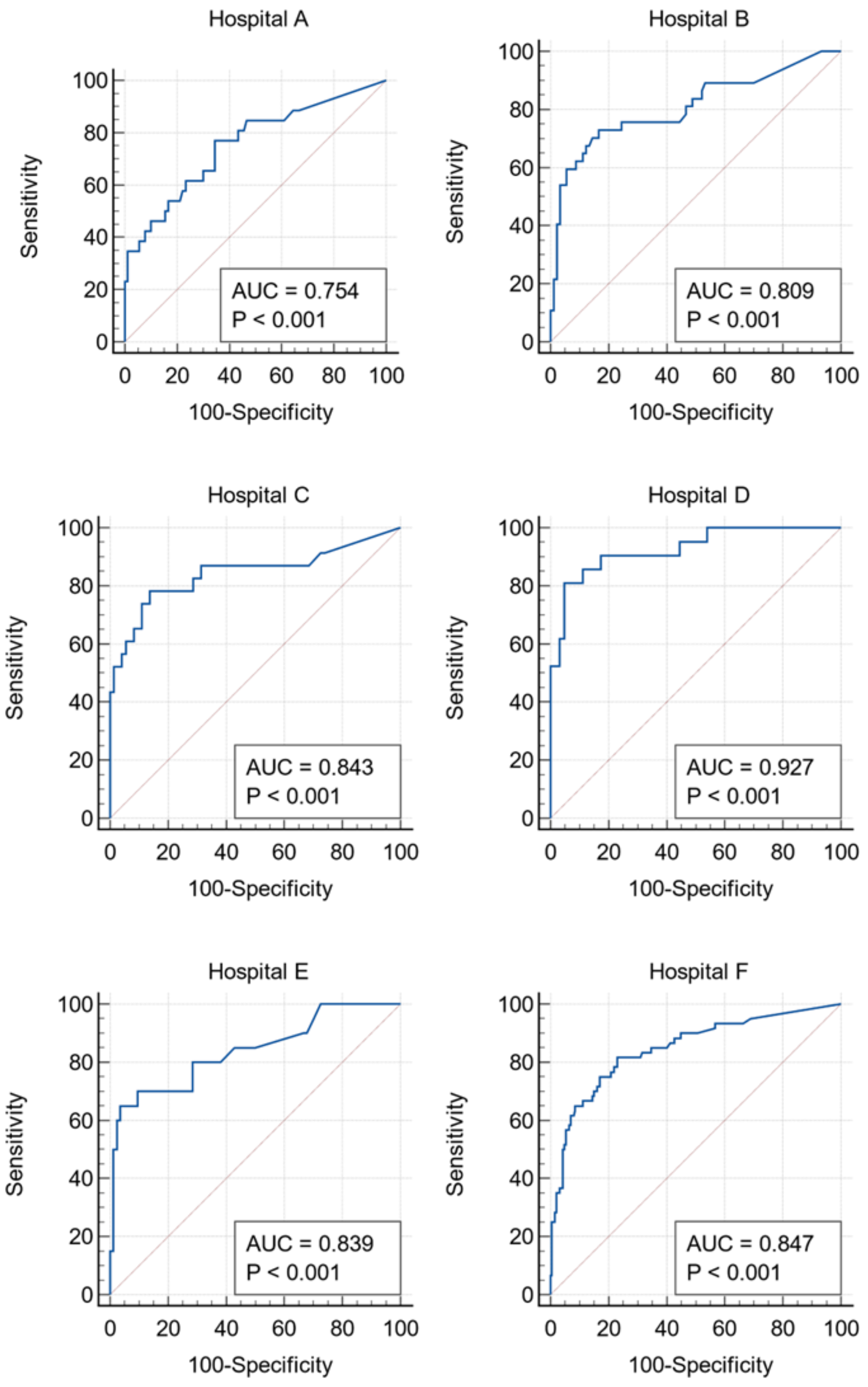

AUC=area under the receiver operating characteristics curve

Supplemental Figure 4. Predicted and observed probabilities of large vessel occlusion (LVO) after stratifying deciles of LVO score and categorized LVO scores

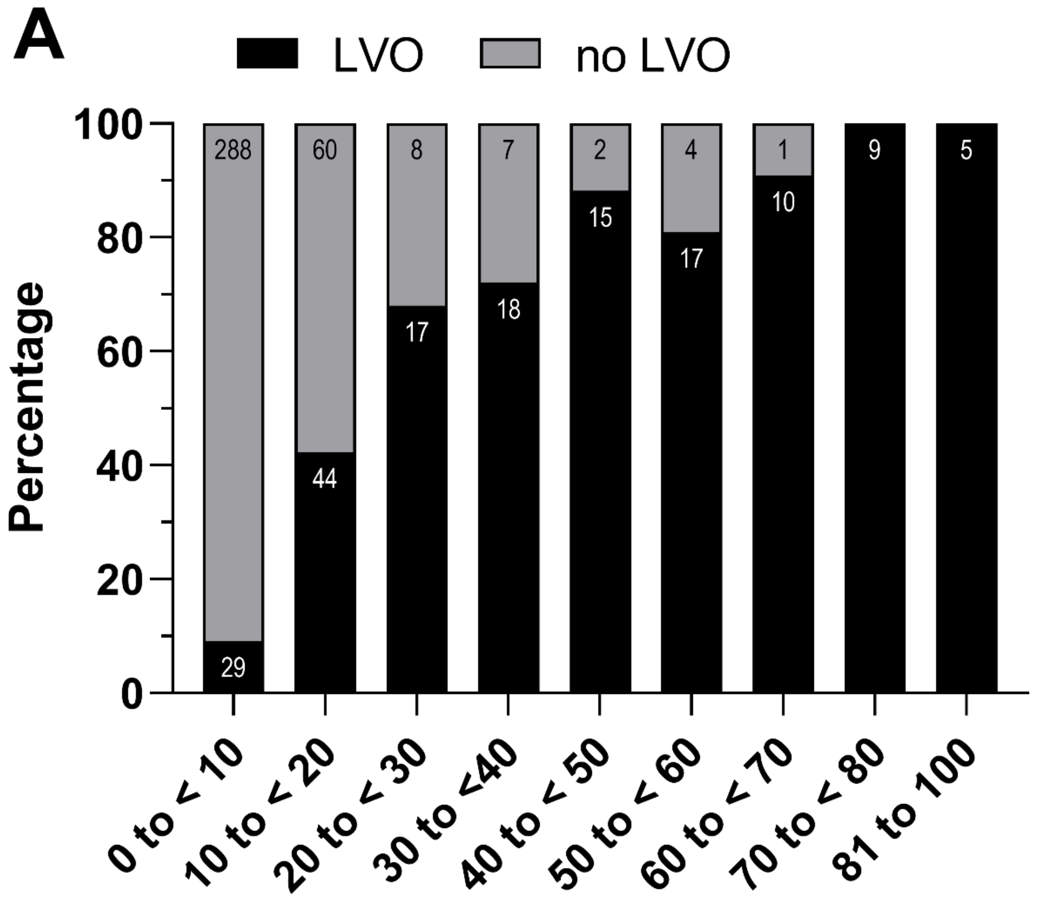

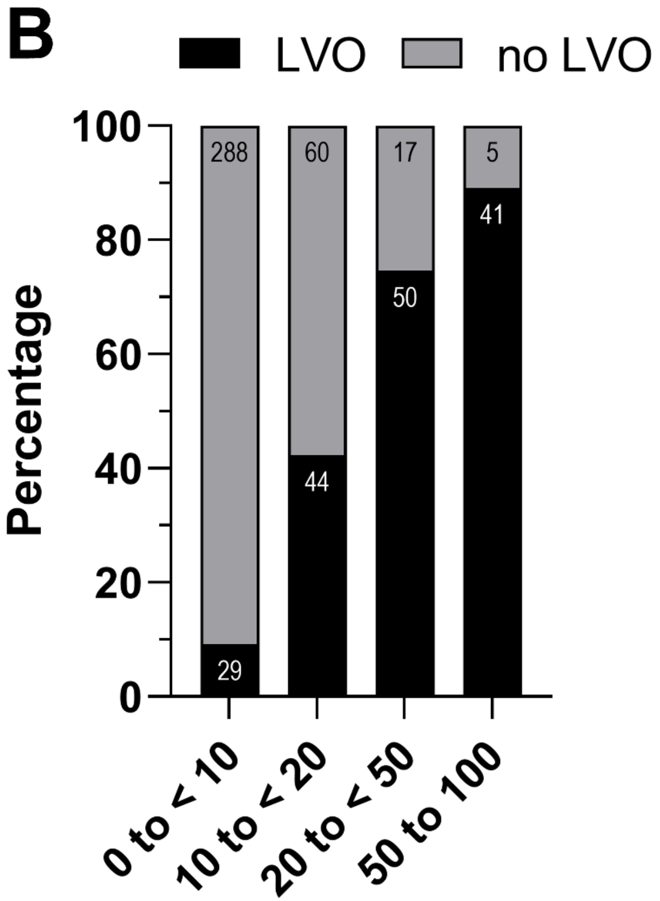

Supplemental Figure 5. Representative case showing the results of JLK-CTL

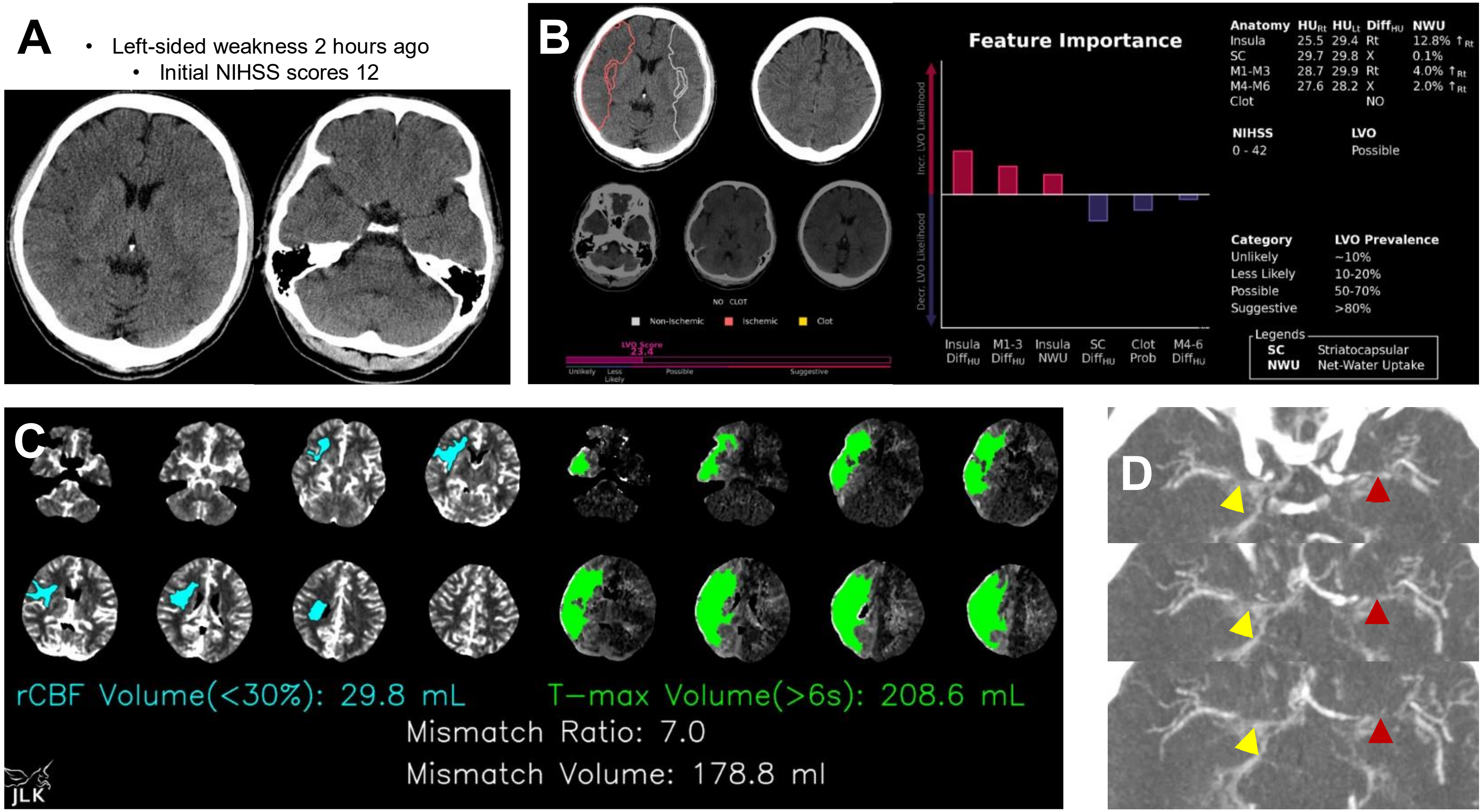

(A) Patient in their 60s presented with left-sided weakness, 2 hours after onset. Initial noncontrast CT scans showed low attenuation in the territory of the middle cerebral artery (MCA) without a visible hyperdense arterial sign. (B) The JLK-CTL detected reduced HU in the insula, M1-M2, and M4-M6 areas, suggesting a possible LVO (50-70%). (C) A CT perfusion scan showed a large infarct core-perfusion mismatch. (D) Concurrent CT angiography revealed occlusion of the right ICA and MCA, along with severe stenosis of the left M1-MCA.

Supplemental Table 1. Noncontrast CT and CT angiography parameters in participating centers

	Hospital A (n = 73)	Hospital B (n = 97)	Hospital C (n = 53)	Hospital D (n = 65)	Hospital E (n = 70)	Hospital F (n = 176)
CT model	iCT 256 (n = 73)	IQon - Spectral CT (n = 2)<br>Revolution CT (n = 95)	SOMATOM Definition Flash (n = 13)<br>SOMATOM Definition Edge (n = 3)<br>SOMATOM Definition AS+ (n = 37)	SOMATOM Force 1F_B (n = 39)<br>SOMATOM Definition Edge (n = 23)<br>Aquilion (n = 2)<br>Aquilion Lightning (n = 1)	Aquilion PRIME (n = 70)	Discovery CT750 HD (n= 170)<br>LightSpeed VCT (n = 3)<br>Revolution Frontier (n =3)
NCCT Thickness, mm	5	5	4	4	3	5
NCCT kVP	120	140	120	120	120	120
NCCT data collection diameter	500	320	500	500	240, 320	320
NCCT current	100 (76 – 100)	302 (289 – 311)	460.5 (402.5 – 506)	147 (147 – 149)	250	300
NCCT convolutional kernel	UC	STANDARD	H30f, Hr40f	H30s, Hr38s, Hr40s	FC68	STANDARD
NCCT rows	512	512	512	512	512	512
NCCT pixel spacing	0.41/0.41	0.37/0.37~0.52/0.52	0.40/040~0.56/0.56	0.36/0.36~0.49/0.49	0.35/0.35~0.52/0.52	0.41/0.41~0.49/0.49
CTA Slice thickness, mm	1.5	0.625	0.75	1.0	2	1.25
CTA kVp	120	120	120	90, 100, or 120	120	120
CTA rotation time,[a] s	0.33	0.5	NA	NA	0.5	0.6

Total Collimation Width, mm	40	40	38.4	38.4	40	40
Spiral Pitch Factor	0.515 and 0.601	0.984375	1.2	0.45, 0.7, and 1.0	0.813	0.984375
mAs	140 and 200	249.5 ~ 251.5	327 ~ 338.5	61.75 ~ 330	100 and 125	60 ~ 376.2

[a]Rotation time was not available in SIEMENTS CT scanners.

NCCT=noncontrast CT; CTA=CT angiography

Supplemental Table 2. Sensitivity of the algorithm after stratification by the site of occlusion

	Site of occlusion		
	ICA	MCA-M1	MCA-M2
True positive	32	77	21
False negative	4	15	16
Sensitivity (95% CI)	88.9% (73.9–96.9%)	83.7% (74.5–90.6%)	56.8% (39.5–72.9%)

ICA=internal carotid artery; MCA=middle cerebral artery; CI=confidence interval.

Supplemental Table 3. Performance of the algorithm after stratification by sex

Confusion matrix	Men (n = 312)		Women (n = 222)	
	Prediction		Prediction	
	LVO	No LVO	LVO	No LVO
Ground truth, LVO	70	22	59	13
Ground truth, no LVO	32	188	30	120
Sensitivity (95% CI)	0.761 (0.661–0.844)		0.819 (0.711–0.900)	
Specificity (95% CI)	0.855 (0.801–0.898)		0.800 (0.727–0.861)	
PPV (95% CI)	0.686 (0.587–0.775)		0.663 (0.555–0.760)	
NPV (95% CI)	0.895 (0.846–0.933)		0.902 (0.839–0.947)	
AUROC	0.848 (0.794–0.902)		0.871 (0.820–0.922)	

LVO=large vessel occlusion; CI=confidence interval; PPV=positive predictive value; NPV=negative predictive value; AUROC=area under the receiver operating characteristics curve

Supplemental Table 4. Details of cases with false positives by the algorithm

Classification	Number (percentage)
Chronic infarct	11 (17.7%)
Calcification of middle cerebral artery	10 (16.1%)
Relevant artery stenosis	9 (14.5%)
Distal vessel (M3 or M4) occlusion	8 (12.9%)
Large infarct without large vessel occlusion	7 (11.3%)
Severe white matter hyperintensity	5 (8.1%)
Artifact	3 (4.8%)
Stent in middle cerebral artery	2 (3.2%)
Occlusion of proximal middle cerebral artery	1 (1.6%)
Unknown	6 (9.7%)

Supplemental Table 5. Specificity of artificial intelligence software by different CT vendors and models in subjects without ischemic stroke

Vendors	Philips		SIEMENS			
Model Name	Brilliance 64 (n=149)		SOMATOM Definition Edge (n=113)		SOMATOM Definition AS+ (n=12)	
Confusion Matrix	Prediction		Prediction		Prediction	
	LVO	No LVO	LVO	No LVO	LVO	No LVO
Ground truth, no LVO	11	138	12	101	3	9
Specificity (95% CI)	0.925 (0.904 – 0.946)		0.893 (0.871 – 0.915)		0.750 (0.630 – 0.876)	

LVO=large vessel occlusion; PPV=positive predictive value; NPV=negative predictive value.

Supplemental Table 6. Multivariable ordinal and binary logistic regression analysis between 3-month functional outcome and JLK-CTL LVO scores

	Ordinal logistic		Binary[a] logistic	
	Adjusted odds ratio (95% CI)	p	Adjusted odds ratio (95% CI)	p
**LVO score, per 1 score**	1.02 (1.01 – 1.03)	0.007	1.02 (1.00 – 1.04)	0.016
Age	1.03 (1.02 – 1.04)	< 0.001	1.04 (1.02 – 1.06)	< 0.001
Sex, male	0.81 (0.59 – 1.13)	0.21	0.55 (0.35 – 0.86)	0.009
Admission NIHSS score	1.17 (1.13 – 1.22)	< 0.001	1.19 (1.13 – 1.25)	< 0.001
Previous stroke	1.80 (1.20 – 2.69)	0.005	2.47 (1.43 – 4.24)	0.001
Hypertension	0.97 (0.68 – 1.37)	0.85	1.30 (0.78 – 2.18)	0.32
Diabetes	1.54 (1.10 – 2.17)	0.012	1.66 (1.03 – 2.66)	0.036
Atrial fibrillation	0.95 (0.65 – 1.39)	0.79	1.05 (0.62 – 1.77)	0.86
Revascularization				
Intravenous only	0.94 (0.52 – 1.68)	0.83	0.91 (0.41 – 2.05)	0.83
Intra-arterial only	1.04 (0.59 – 1.83)	0.88	1.66 (0.76 – 3.63)	0.21
Combined	0.37 (0.19 – 0.72)	0.003	0.35 (0.14 – 0.86)	0.022
Onset to NCCT scan, per hour	1.01 (1.00 – 1.03)	0.052	1.03 (1.00 – 1.06)	0.076

[a]For binary logistic regression analysis, modified Rankin Scale scores were dichotomized into favorable (0, 1, and 2) vs unfavorable (3 – 6) groups.
LVO=large vessel occlusion; NIHSS=National Institute Health Stroke Scale; NCCT=noncontrast CT

Supplemental Table 7. Multivariable binary logistic regression analysis between binary unfavorable 3-month outcome and JLK CTL LVO scores after stratification by endovascular thrombectomy

	Without EVT (n = 424)		With EVT (n = 110)	
	Adjusted odds ratio (95% CI)	p	Adjusted odds ratio (95% CI)	p
LVO score, per 1 score	1.00 (0.98 – 1.03)	0.70	1.03 (1.01 – 1.06)	0.013
Age	1.04 (1.01 – 1.06)	0.002	1.04 (1.00 – 1.08)	0.080
Sex, male	0.55 (0.33 – 0.93)	0.026	0.58 (0.22 – 1.53)	0.27
Admission NIHSS score	1.23 (1.16 – 1.30)	< 0.001	1.10 (1.00 – 1.22)	0.055
Previous stroke	2.85 (1.57 – 5.16)	0.001	1.26 (0.34 – 4.63)	0.73
Hypertension	1.52 (0.82 – 2.82)	0.18	1.00 (0.37 – 2.67)	1.00
Diabetes	1.33 (0.78 – 2.26)	0.30	3.55 (1.08 – 11.65)	0.037
Atrial fibrillation	1.40 (0.75 – 2.58)	0.29	0.71 (0.26 – 1.97)	0.52
Intravenous thrombolysis	0.89 (0.38 – 2.06)	0.78	0.27 (0.10 – 0.74)	0.011
Onset to NCCT scan, per hour	1.03 (0.996 – 1.07)	0.076	1.02 (0.92 – 1.13)	0.65

[a]For binary logistic regression analysis, modified Rankin Scale scores were dichotomized into favorable (0, 1, and 2) vs unfavorable (3 – 6) groups.

LVO=large vessel occlusion; NIHSS=National Institute Health Stroke Scale; NCCT=noncontrast CT
